# IMPLEMENTING CARE COORDINATION WITH HOME DELIVERED MEALS AND HEALTHCARE: CHALLENGES AND LESSONS LEARNED

**DOI:** 10.1093/geroni/igad104.3423

**Published:** 2023-12-21

**Authors:** Elizabeth Orsega-Smith, Julia O’Hanlon, Allison Karpyn

**Affiliations:** University of Delaware, Newark, Delaware, United States; University of Delaware, Newark, Delaware, United States; University of Delaware, Newark, Delaware, United States

## Abstract

Increasing lifespans are resulting in frail, isolated and food insecure older adults. Home Delivered meals (HDMs) are gateways into the homes of frail and vulnerable older adult, and this program will explore how HDMs can provide a natural opportunity to identify health issues potentially preventing unnecessary emergency rooms visits and hospitalizations. A daily wellness check and care coordination involved the use of a program coordinator, a nurse advocate, a local and predominately rural senior center (HDM provider and its HDM volunteers), and university evaluation team designed to support innovative health services provided to vulnerable older adults. The purpose was to examine the evaluation results 12 months after program. At baseline, clients reported high physical dependence (91.6%), hypertension (72.13% ), risk for falls (100%), and risk of food insecurity (48%). During the 12 month period, the nurse advocate team made 6,096 phone calls to participants with most phone calls (79.5%) categorized as a general client check-ins. Calls directly impacting quality of life included transportation support (6.1%), housing support (4.3%), and access to medical equipment (6.7%). Approximately 650 calls were service-related calls to outside agencies, organizations, and medical providers. This model exemplifies the increasing importance of clinicians and public health professionals, working collaboratively with community-based resources. The role of the nurse advocate team is critical in connecting resources to individuals based on specific circumstances and conditions, including geographic location and ability to access medical and social services. These efforts helped to avoid expensive treatments, manage living challenges, and facilitate medical care.

